# Case Report: Discontinuous Spontaneous Ventilating Anesthesia for McKeown Esophagectomy by Laryngeal Mask: A Case Series—A Novel Approach of Discontinuous Spontaneous Ventilating Anesthesia for Esophagectomy

**DOI:** 10.3389/fsurg.2021.783859

**Published:** 2021-12-09

**Authors:** Qiaoqiao Xu, Xuan Mo, Juan Xiong, Yi Zhang

**Affiliations:** Department of Anesthesiology, Tongji Hospital, Tongji Medical College, Huazhong Science and Technology University, Wuhan, China

**Keywords:** ERAS, LMA, NIVATS, esophagectomy, case report

## Abstract

Double lumen endobronchial ventilation in McKeown esophagectomy is common for esophageal cancer. In spite of most patients could be extubated immediately after surgery under adequate multimodal analgesia, still some patients require extended mechanical ventilation or airway support post-surgery because of pain or difficult respiration. The present study reported a novel challenge for McKeown esophagectomy with discontinuous spontaneous ventilating anesthesia by the laryngeal mask. Three esophageal cancer patients underwent McKeown esophagectomy under discontinuous spontaneous ventilating anesthesia with local and regional analgesia and appropriate sedation. Two of them were accomplished under non-intubated video-assisted thoracoscopic surgery (NIVATS), and then, the abdominal and neck surgery was managed under laryngeal mask airway with appropriate muscle relaxation. One patient was endured high PetCO_2_ level, and converted to regular double lumen endobronchial intubation for safety. However, from the two successful cases, we still proved that the discontinuous spontaneous ventilating anesthesia achieved the same anesthetic effect as bronchial intubation under general anesthesia for McKeown esophagectomy, which reduced the postoperative pharyngeal discomfort, might be beneficial to the patients for enhanced recovery after surgery (ERAS).

## Introduction

Esophageal cancer is a common gastrointestinal malignancy, and McKeown esophagectomy with three incisions is the recommended surgical treatment (1). Double lumen endobronchial ventilation is employed under high-dose intravenous anesthetic, which necessitates the patient to accept extended mechanical ventilation or airway support after the surgery (2). Moreover, the postoperative pharyngeal discomfort caused by intubation is conspicuous (3, 4). These issues hinder the enhanced recovery after surgery (ERAS). If multimodal analgesia can be provided using local and regional analgesia and appropriate sedation, the thoracic operation could be accomplished under spontaneous respiration as non-intubated video-assisted thoracoscopic surgery (NIVATS) for the patient.

Herein, we reported a case series on the discontinuous spontaneous ventilating anesthesia for McKeown esophagectomy by laryngeal mask and evaluated the effect, safety, and feasibility of the anesthesia.

## Case Report

Three patients, with body mass index (BMI) <25 kg/m^2^ and American Society of Anesthesiologists (ASA) I-II, were admitted with progressive dysphagia. These three patients were diagnosed as esophageal cancer without any distinct cardiovascular disease. Airway evaluation revealed normal open mouth, Mallampati II, thyromental distance >6.5 cm, and no limitation of cervical activity. Two patients underwent surgeries under discontinuous spontaneous ventilating anesthesia; the remaining patient was converted to regular double lumen endobronchial intubation due to high PetCO_2_ level. Before the operation, the patients were informed about the anesthesia approach, and signed consents were obtained for the publication of this report.

## Anesthesia Management

### Induction of Anesthesia

On the operation day, the patients were fasted over 8 h and were not administered preoperative medication before anesthesia. Electrocardiogram, blood pressure, and pulse oxygen saturation (SpO_2_) were monitored when the patients entered the operating room. Intravenous access was established, the left radial artery puncture catheterization was administered, and the pressure was measured. In addition, 6 L/min oxygen was administered for 3 min by face mask.

Preparation for anesthesia respiratory management: Double-tube laryngeal mask (Supreme), double-lumen tube, single tracheal tube, laryngoscope, and aspirator were prepared. General anesthesia was induced by sufentanil 10 μg, dexmedetomidine 0.5 μg/kg/h. After 10 min, 2 mg/kg propofol was administered intravenously to make the patients unconscious. Then, a suitable size laryngeal mask was inserted without the help of a muscle relaxant, followed by simultaneous intermittent mandatory ventilation mode. Subsequently, the anesthesia was maintained with sevoflurane 0.8–1.0%, dexmedetomidine 0.5 μg/kg/h, remifentanil 0.01–0.05 μ g/kg/min, and propofol 3–5 mg/kg/h. The bispectral index (BIS) value was 40–60. Finally, the central venous catheter was inserted under ultrasonic guidance.

### Nerve Block Method

Bilateral transverse abdominal plane (TAP) nerve block was administrated using 20 mL of 0.4% ropivacaine. Then, the patient was laid on the left side to administer the right-side vertebral nerve block (T5/6, T7/8) using 7 mL of 0.4% ropivacaine at each point. The surgeon selected the T5 hole on axillary midline for operation, and T8 hole on the posterior axillary line for observation. One of the patients did not receive cervical plexus block for the first attempt, but the other two patients were administered left cervical plexus block by 2 mL of 0.4% ropivacaine before cervical surgery. All the nerve block administrations were guided by ultrasound. During the surgery, the thoracic surgeon opened a 3 cm incision on the T5 intercostal on the right axillary midline. Gradually, the patients recovered spontaneous respiration and the spontaneous respiration was stable. The right lung collapsed gradually under atmospheric pressure (same as artificial pneumothorax). Then, the surgeon sprayed 10 mL of 1% lidocaine and 0.5% ropivacaine mixture to the pleural surface, the right vagus nerve, and the phrenic nerve block under the guidance of a thoracoscope.

### Surgical Progress

There were three periods according to the incision sites.

#### The First Period: NIVATS Anesthesia Reserving Spontaneous Respiration

**Figure 1:**
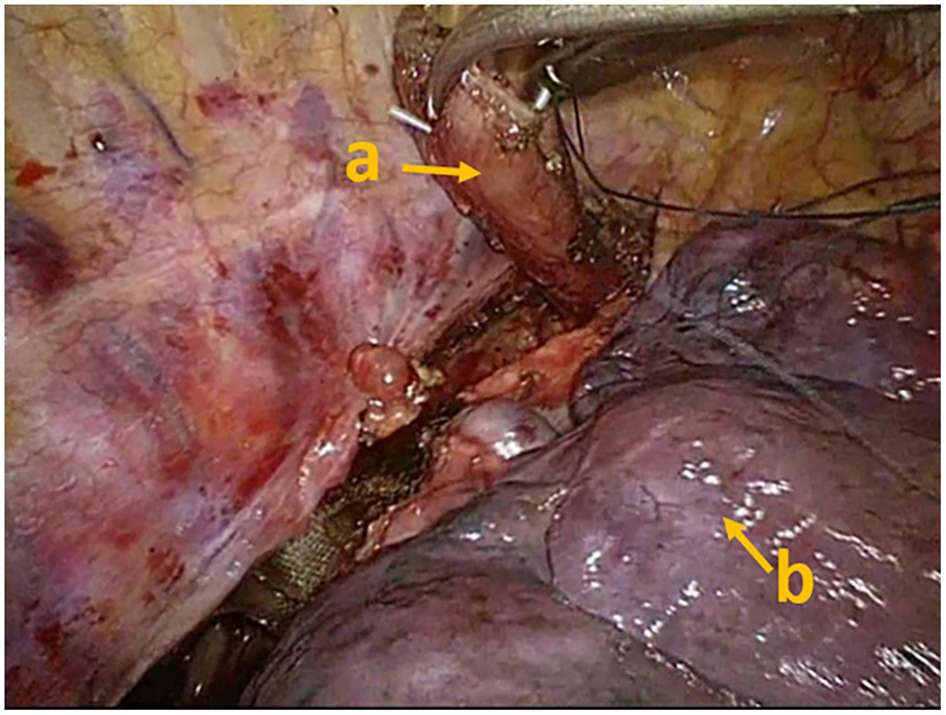
The thoracic section of the esophagus was dissociated, which could avoid the risk of esophageal reflux. **(a)** Esophageal stump, **(b)** pulmonary lobe.

The dose of remifentanil was adjusted to 0.01–0.05 μg/kg/min to maintain the appropriate spontaneous respiration frequency (10–15 times/min) and the appropriate tidal volume of 200–300 mL, thereby reducing the mediastinal oscillation. The adjusted remifentanil dosage, reduced the respiratory frequency and tidal volume, even the respiration stopped to make the lung “silent” for a few minutes providing an optimal surgical field for the surgeon to separate the lesions, reduce the bleeding, and avoid the misidentification of large blood vessels. It could lead to the accumulation of carbon dioxide, i.e., transient permissive hypercapnia (PHY). The thoracic section of the esophagus was dissociated at 5 cm proximal to the tumor, and then, the lymph nodes of each station were cleared, showed in Figure 1. After sputum suction and lung dilation, the thoracic sectioning was accomplished. The PetCO_2_ level was maintained at 47–62 mmHg during this period in these two successful patients. However, one of the patients was transit to double-lumen endobronchial intubation because the PetCO_2_ level was high (>75 mmHg). For safety, the remaining surgery was carried out using the regular double-lumen endobronchial ventilation anesthesia as the muscle relaxant.

#### The Second Period: Abdominal Surgery Anesthesia in These Two Successful Patients

After the thoracic segment surgery, 2 mg/kg cisatracurium (non-depolarizing agent) was utilized for interrupted spontaneous respiration, which was converted to mechanical ventilation. The abdominal operation process was chosen the tube creation through mini-laparotomy with short CO_2_ pneumoperitoneum time. And CO_2_ pneumoperitoneum should be ensured the pneumoperitoneum pressure was controlled <12 mmHg, and the PetCO_2_ level was maintained at 42–49 mmHg.

#### The Third Period: Cervical Surgery Anesthesia in These Two Successful Patients

Cervical surgery was performed by facilitating an incision in the left cervical sternocleidomastoid muscle. The cervical esophagus was dissociated, and the anastomosis of the tubular stomach and esophagus was achieved. Then, the surgeon gently pulled out the trachea in case the laryngeal mask was displaced. In order to adjust the position of the laryngeal mask, the appropriate small dose of the muscle relaxant was used to reduce the displacement or leakage of the laryngeal mask. The PetCO_2_ level was maintained at 41–48 mmHg in this period.

#### Anesthesia Recovery in These Two Successful Patients

During the cervical and abdominal incision closure process, the spontaneous respiration recovered in these patients without CO_2_ accumulation, and the consciousness was recovered after 10 min. One of the patients awoke immediately, and the muscle strength was restored after the laryngeal mask was pulled out. The patient was able to move to the transport bed without aid, and then sent to the post-anesthesia care unit (PACU). The second patient was dysphoric during anesthesia recovery, which might be attributed to the sevoflurane remnant. The last patient received double-lumen endobronchial intubation was extubated and awoke immediately as well. After the surgery, no adverse reactions and complications were detected in these three patents, and the blood routine examination, blood biochemical examination, and normal iodine water radiography were reviewed. The leucocyte level of the intubated patient was higher and the throat discomfort was much more severe than that in the other two patients. The perioperative clinical data of these patients was summarized in Table 1. Then, the chest tube and stomach tube were removed, and the food intake was restored gradually. The patients were discharged on day 11.

**Table 1:** Perioperative clinical data of the three patients.

**Variable**	**Case 1**	**Case 2**	**Case 3**
Gender, M/F	M	M	M
Age (years)	53	64	54
BMI (kg/m^2^)	23.72	21.63	22.32
Concomitant disease	No	No	No
Airway tools	LMA	LMA	LMA-DLT
Thoracic surgery (min)	90	70	120
Abdominal surgery (min)	95	75	70
Cervical surgery (min)	95	75	70
Infusion quantity (mL)	3,950	4,000	4,000
Blood loss volume (mL)	200	200	250
Urine volume (mL)	1,200	1,000	600
Leucocyte level (POD 1, × 10^9^/L)	9.21	7.16	7.31
Leucocyte level (POD 2, × 10^9^/L)	13.05	14.96	17.96
Leucocyte level (POD 3, × 10^9^/L)	9.17	8.18	12.16
The throat discomfort	Mild	Mild	Serious
The iodine water radiography time (POD)	10	10	10
Discharge date (POD)	11	11	11

*M, male; F, female; BMI, body mass index; LMA, laryngeal mask airway; DLT, double-lumen tube; POD, postoperative day*.

## Discussion

General anesthesia using double-lumen endotracheal intubation with one-lung positive pressure ventilation and muscle relaxants is the regular method for thoracic surgery. However, the potential complications related to tracheal intubation and postoperative pharyngeal discomfort caused by intubation are conspicuous. Professor Eugenio Pompeo et al. first proposed the non-tracheal intubation technique to support thoracic surgery (5, 6). Furthermore, NIVATS natural airway anesthesia methods could be used in the pulmonary bullae resection, lobectomy, bronchoplasty, angioplasty, or tracheal (7, 8).

Multimodal analgesia for McKeown esophagectomy with three incisions could be implemented as NIVATS, which was accomplished under spontaneous respiration, followed by abdominal and neck surgeries after muscle relaxation. This bold attempt was mainly due to the use of double-lumen laryngeal mask. If the suitable position and good sealing of the laryngeal mask were sure, it could provide the isolation and aspiration of oral secretions, and ensure the safety of patients during switching between spontaneous respiration and mechanical ventilation. In the period of laparoscopic operation, the severed esophagus completely avoids the risk of reflux and aspiration which compensated for the shortcoming of the laryngeal mask. The neck surgery was influenced by the laryngeal mask, but Cherie P. Erkmen utilized the pig model to demonstrated that the cervical esophageal anastomosis of esophageal surgery was tolerable and the pressure was higher than the non-invasive ventilation pressure of the laryngeal mask, which would not lead to complications such as leakage (9, 10). This phenomenon hinted that laryngeal mask airway is feasible in the intraoperative cervical anastomosis, which would exert the anesthesia effect similar to that of bronchial intubation under general anesthesia with reduced postoperative pharyngeal discomfort and early ambulation after the operation.

Compared to the conventional double-lumen endotracheal intubation anesthesia in esophagectomy, anesthetics were less in these cases, and airway injury caused by endotracheal intubation was avoided by the use of laryngeal mask, reducing the respiratory complications of mechanical ventilation and the side effects of muscle relaxants. Meanwhile, it provided the stable hemodynamics and the postoperative analgesia achieved the whole cover for three incisions by nerve block. In our cases, the leucocyte level of the intubated patient was higher and the throat discomfort was much more severe than that in the other two patients. Even though early extubation would be possible after the surgery immediately, the side eff effects of the mechanical ventilation, inflammatory factor, and discomfort could not be avoided. Dr. He's group has compared the impact of non-intubated *vs*. intubated anesthesia on the early outcomes of video-assisted thoracoscopic anatomical resection in lung cancer, showed that the postoperative fasting time, the overall postoperative chest drainage volume, and the length of hospital stay were improved significantly in the NIVATS group (11, 12). Then Dr. He's group systematically assessed the feasibility and safety of non-intubated VATS compared with intubated VATS perioperatively for the treatment of different thoracic diseases by extensive search of literature databases, and concluded that non-intubated VATS may be a safe and feasible alternative to intubated VATS and provide a more rapid postoperative rehabilitation time than conventional intubated VATS (13).

The key points of the non-tracheal intubation anesthesia in three incisions of McKeown esophagectomy were as follows: (1) The establishment and maintenance of spontaneous respiration of thoracic surgery; (2) The application of the laryngeal mask in laparoscopic surgery and the requirement of the loosened abdominal muscles; (3) The ensured position of the laryngeal mask in the cervical throat surgery period. The difference in the thoracic surgery in these cases and conventional NIVATS was the requirement of clearer vision and smaller mediastinal swing to avoid hindering the operation or cause hemorrhage to the vessels that would result in intubation and thoracotomy. Using short-term effect analgesic remifentanil, alleviated the respiratory amplitude, which was advantageous to the surgery, but also, allowed carbon dioxide retention. Therefore, the anesthesiologist needs to balance the accurate dose (14). In addition, the maintenance of left side lung ventilation by spontaneous respiration depends on the integrity of the pleural cavity, which should not break during esophagus and lymph node dissection.

The indications and contraindications, such as cardiovascular complications, pulmonary dysfunction, obesity (BMI >30 kg/m^2^), foreseeable difficult airway, prolonged surgery, weak surgical team, inexperienced anesthetist or team should be cautious for non-tracheal intubation anesthesia and discontinuous spontaneous ventilation in McKeown three incisions esophagectomy (15). Furthermore, in the case of high PetCO_2_ level and massive hemorrhage, endotracheal intubation should be converted to general anesthesia immediately.

Our group was one of the first to apply non-tracheal intubation anesthesia in three incisions esophageal cancer surgery as early as in 2018. And many McKeown esophagectomy using discontinuous spontaneous ventilating anesthesia was repeated after that, based on these three cases experience. It was reported that minimally invasive esophagectomy was managed under laryngeal mask airway general anesthesia, which was mutual corroborate with our attempt (16). However, it was minimally invasive esophagectomy, the incision and complexity were much simpler than McKeown three incisions esophagectomy in our cases.

Taken together, we accomplished a pioneer study about non-tracheal intubation anesthesia and discontinuous spontaneous ventilation in three incisions McKeown esophagectomy. With this procedure, the side effects of the regular double-lumen endobronchial ventilation anesthesia could be reduced. The limitation was that it was only 3 patients enrolled, and in one case the recommended procedure was not successful. From these failure cases and exploratory stage, it reminded us more experience for better perioperative management and safety. And the optimized procedure could be popularized and expanded the treatment scope in the NIVATS anesthesia.

## Data Availability Statement

The original contributions presented in the study are included in the article/supplementary material, further inquiries can be directed to the corresponding authors.

## Ethics Statement

Written informed consent was obtained from the individual(s) for the publication of any potentially identifiable images or data included in this article.

## Author Contributions

QX was responsible for reviewing publications and drafting of the manuscript. XM was responsible for data curation. JX was responsible for drafting of the manuscript. YZ was responsible for the conception of the review, supervision, and critical revision of the manuscript. All authors contributed to the article and approved the submitted version.

## Conflict of Interest

The authors declare that the research was conducted in the absence of any commercial or financial relationships that could be construed as a potential conflict of interest.

## Publisher's Note

All claims expressed in this article are solely those of the authors and do not necessarily represent those of their affiliated organizations, or those of the publisher, the editors and the reviewers. Any product that may be evaluated in this article, or claim that may be made by its manufacturer, is not guaranteed or endorsed by the publisher.
